# A Biomimetic Adhesive/Kaolin Material with Strong Adhesion, Sealing, and Active Coagulation Function for Arterial Hemostasis

**DOI:** 10.3390/ijms26104688

**Published:** 2025-05-14

**Authors:** Wanli Zhang, Junqin Mao, Aiping Yang, Tao Shen, Qiuyu Zeng, You Tang, Yan Du, Guoyu Lv, Heng Zheng, Hong Li

**Affiliations:** 1 College of Physics, Sichuan University, Chengdu 610065, China; 2 College of Information Engineering and Automation, Kunming University of Science and Technology, Kunming 650032, China

**Keywords:** arterial hemorrhage, biomimetic adhesive, kaolin, strong adhesion hemostasis

## Abstract

Effective prehospital hemostasis is pivotal for improving survival outcomes in arterial hemorrhage. Existing hemostatic materials have limitations in terms of both efficiency and portability, particularly under complex wound conditions. This study engineered a kaolin-reinforced biomimetic adhesive composite powder (DTG-K), which achieved dual hemostatic mechanisms: mechanical occlusion via strong interfacial adhesion (lap-shear strength of 58.3 kPa) and biochemical activation of the intrinsic coagulation pathway through factor XII. In vitro evaluations confirmed its exceptional hemocompatibility, with a 0.93% hemolysis rate. When applied to femoral artery hemorrhage models in Bama swine, DTG-K demonstrated complete hemostasis within 30 s through synergistic physical sealing and biochemical coagulation. These findings demonstrate the potential of DTG-K as an innovative strategy for managing life-threatening arterial hemorrhage in prehospital emergency scenarios.

## 1. Introductions

Arterial hemorrhage is one of the leading causes of death in combat injuries, traffic accidents, and surgical procedures. Severe bleeding accounts for over 35% of prehospital deaths and nearly 40% of deaths within 24 h post injury [1]. For instance, rupture of the femoral or carotid artery can result in a hemorrhage rate of up to 800 mL in 30 s, meaning that an adult patient may enter hemorrhagic shock within 30 s, requiring the wound to be treated within a few minutes. Existing clinical hemostatic materials primarily rely on physical tamponade (e.g., gauze) or coagulation factor activation (e.g., zeolite or kaolin) [2]. However, in high-pressure arterial bleeding scenarios, these materials are often washed away by the high-velocity blood flow and struggle to balance hemostatic efficiency with tissue compatibility [3]. For example, the kaolin-impregnated combat gauze (combat gauze) widely used by the U.S. military achieves rapid hemostasis by activating coagulation factor XII [4]; however, its reliance on multilayer wrapping and compression limits its application in deep wounds or body cavity hemorrhages. Therefore, the development of multifunctional hemostatic materials that combine rapid adhesion and sealing, active coagulation activation, and biosafety has become an urgent need in the field of emergency medicine.

Aggregating hemostatic materials have shown significant potential in the treatment of complex traumatic injuries through the dual mechanisms of interfacial adhesion and blood component activation. For example, chitosan-based materials can adsorb negatively charged red blood cells and platelets through electrostatic interactions due to their positively charged surface, forming a physical aggregation barrier. The HemCon^®^ bandage used by the U.S. military is based on this principle and has achieved rapid hemostasis in battlefield first aid [5,6,7]. However, in cases of high-pressure arterial bleeding (>100 mL/min), this material requires external compression and is prone to being washed away by blood flow due to insufficient interfacial adhesion [3]. Similarly, fibrin glue forms a fibrin network by mimicking the terminal reaction of the coagulation cascade. Although it has excellent biocompatibility, its adhesion strength on wet tissue surfaces is weak (<10 kPa); animal-derived components may trigger immune rejection [8]. The chitosan-oxidized bacterial nanocellulose composite material developed by Donghua University—which is formed into a porous sponge structure via freeze-drying technology—was able to shorten the clotting time to 1.5 min in a rat liver model [9]. However, it is at risk of structural collapse under the impact of dynamic blood flow. These cases indicate that a single aggregation mechanism is insufficient to meet the clinical demands of arterial hemostasis in high-pressure and wet environments.

To address these challenges, polymers with catechol-based groups, inspired by mussel foot proteins, have become a research focus for next-generation hemostatic materials due to their unique wet adhesion properties to tissue. Mussels secrete catechol-based groups from their foot proteins, which can form multiple non-covalent bonds (hydrogen bonds, π-π stacking, and metal coordination) with amino and hydroxyl groups on tissue surfaces. After oxidation, covalent crosslinking is formed, achieving high-intensity underwater adhesion (>50 kPa) [10,11,12,13]. Based on this, previous studies have developed various biomimetic adhesive materials. For example, Han et al. prepared a hydrogel with a wet adhesion strength of 34 kPa on pigskin surfaces using a polydopamine–clay composite strategy [14]. Ai’s team combined catechol-modified acrylamide monomers with thermosensitive materials to develop a photocurable biological glue [15]. However, this glue lacks active hemostatic functions; its hemostatic efficiency depends on the host’s own coagulation ability. In particular, there are no reports on the application of hemostatic materials to a femoral artery hemorrhage model. It should be noted that most existing biomimetic adhesives focus on optimizing adhesion performance while neglecting active regulation of the coagulation cascade. This results in limited efficacy in scenarios with coagulopathy or high blood flow rates.

Therefore, this study proposes incorporating kaolin into a catechol-based biomimetic adhesive system to construct a composite hemostatic material with synergistic “adhesion–coagulation” enhancement. Kaolin, a natural silicate mineral, has a lamellar structure that not only forms hydrogen bonds with polymer networks through surface hydroxyl groups to enhance material cohesion, but can also activate coagulation factor XII via its negative charge, accelerating the conversion of fibrinogen to fibrin and thereby accelerating the coagulation process [2]. This design may overcome the limitations of the existing hemostatic materials with single-action mechanisms: on the one hand, the catechol groups enable rapid anchoring to moist wound surfaces while resisting being washed away by blood flow. At the same time, the adhesion to tissue can rapidly seal the bleeding wound. On the other hand, the kaolin-driven activation of coagulation accelerates the hemostatic process, achieving active hemostasis and reducing the reliance on the body’s intrinsic blood-coagulation function. Through systematically optimizing the kaolin doping ratio and interfacial interactions, this study aimed to develop an arterial hemostatic material with high-pressure adaptability, rapid responsiveness, and biosafety, providing a new hemostatic agent for the treatment of casualties with arterial bleeding before evacuation.

## 2. Results

### 2.1. Chemical Characterization Results

**Figure 1:**
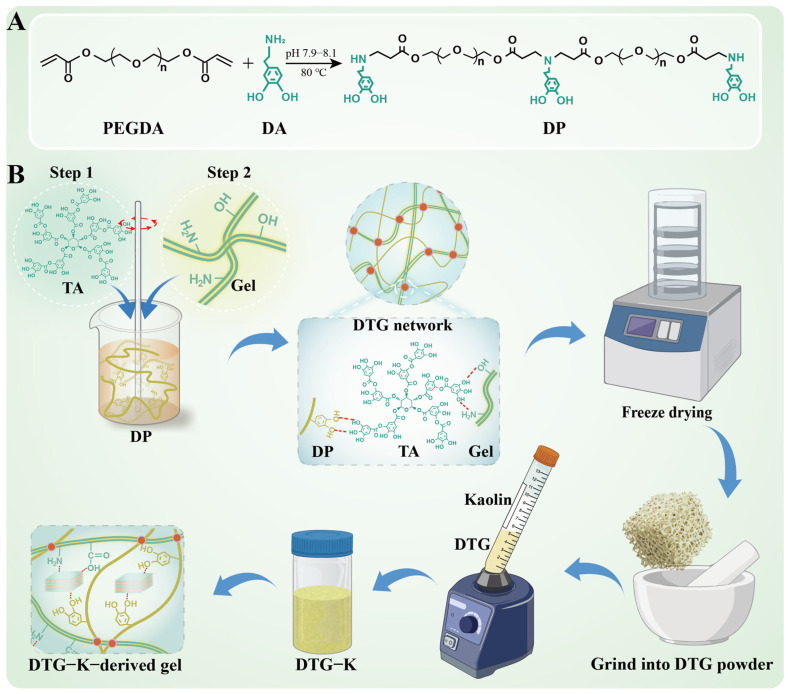
(**A**) Preparation of catechol-functionalized polymer DP; and (**B**) preparation of DTG-K.

The synthesis of the biomimetic adhesive/kaolin material is shown in Figure 1. In the material design, dopamine hydrochloride (DA) provides the biomimetic adhesive with catechol groups for tissue adhesion; it copolymerizes with polyethylene glycol diacrylate (PEGDA) to form DP (derived from the initials of DA and PEGDA), one of the backbones that is primarily responsible for the adhesion. Gelatin (gel) serves as an additional framework for the biomimetic adhesive material, intercrossing and crosslinking with DP. Tannic acid (TA) acts as a crosslinking agent for both the gel and DP. Kaolin is added as an enhancer of cohesion and an activator of coagulation mechanisms.

Dopamine hydrochloride (DA) showed IR absorption peaks at 3336.4 cm^−1^ (N-H) and 1284.4 cm^−1^ (phenolic hydroxyl; Figure S1, Supporting Information). Poly (ethylene glycol) diacrylate (PEGDA) showed peaks at 1635.4/1616.9 cm^−1^ (C=C), 2865.8 cm^−1^ (C-H), and 1722.2/1097.3 cm^−1^ (C=O and C-O-C), indicating its double-bond and ester structures. In DP’s spectrum, the C=C peaks disappeared, while new peaks emerged at 1731.8 cm^−1^ (C=O shift) and 1016.3 cm^−1^ (C-O-C low-frequency shift); the phenolic hydroxyl peak (1284.4 cm^−1^) remained. This confirmed that the C=C bonds in PEGDA underwent Michael addition with DA’s amino groups, forming a catechol-terminated alternating copolymer DP (derived from the initials of DA and PEGDA). The ^1^H NMR analysis further confirmed this (Figure S2, Supplementary Materials): DP’s spectrum retained DA’s aromatic proton signals (δ 6.70–6.41 ppm) and PEGDA’s ether methylene signals (δ 3.75–3.15 ppm). The vinyl signals (δ 6.40–5.85 ppm) disappeared; new methylene peaks adjacent to secondary amines (δ 3.45–3.10 ppm) emerged, verifying the formation of the alternating copolymer DP [16,17,18,19].

DTG (derived from the initials of DP, TA, and gel) is a gel-forming agent that was synthesized by mixing DP, a gelatin (gel) solution, and a tannic acid (TA) solution. Its crosslinking depends on the hydrogen bonding between TA’s phenolic hydroxyl groups and DP and gel. The TA showed IR peaks at 1444.4/1309.4 cm^−1^ (C-O/-OH) and 1697.1 cm^−1^ (C=O) (Figure S3, Supplementary Materials). The gel had peaks at 3278.5 cm^−1^ (N-H), 1525.4 cm^−1^ (C-N), and 1627.7 cm^−1^ (C=O) due to its amino and carboxyl groups. The analysis of DTG’s IR spectrum revealed a low-frequency shift of the O-H peak at 3150 cm^−1^ and a shift of the C=O peak at 1716.4 cm^−1^ compared to DP, both attributed to hydrogen bonding with hydroxyl groups. The -OH shift at 1313.3 cm^−1^ also supports these results [20]. TA’s characteristic peak at 754.1 cm^−1^, likely due to the out-of-plane bending of C-H in the benzene ring, was retained. Additionally, the absorption peaks at 1606.4 cm^−1^ (C-O from phenolic hydroxyl groups) and 1444.4 cm^−1^ (C=C from the benzene ring structure) were preserved.

**Figure 2:**
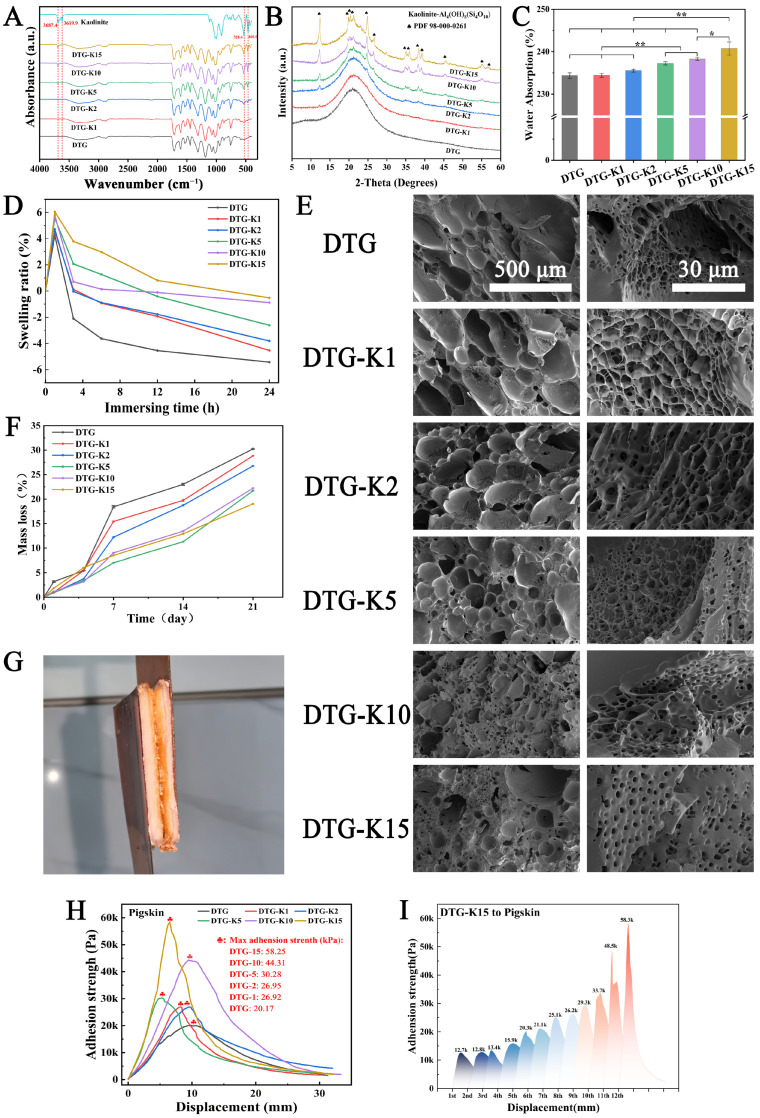
(**A**) FTIR spectra of DTG, kaolin, and DTG-K materials after secondary lyophilization following rehydration; (**B**) XRD patterns of DTG-K materials after secondary lyophilization following rehydration; (**C**) water absorption of DTG and DTG−K powders; (**D**) swelling ratio of DTG and DTG-K powders (n = 3, * *p* ≤ 0.05, ** *p* ≤ 0.01); (**E**) SEM images of DTG- and DTG-K-derived gels after lyophilization; (**F**) in vitro degradation curves of DTG- and DTG-K-derived gels; (**G**) schematic of overlap–shear adhesion tests on pigskin; (**H**) adhesion strength of DTG- and DTG-K-derived gels on pigskin; and (**I**) adhesion strength of DTG-K15-derived gel in repeated pigskin adhesion tests.

The DTG powder, kaolin powder, and re-lyophilized DTG-K (derived from DTG and the initial of Kao) powders were characterized using FTIR and XRD analyses. The FTIR spectra (Figure 2A) showed shifts in kaolin’s Al/Si-OH peaks (3693.2, 914.2, and 1006.7 cm^−1^) and DTG’s -CH2-NH2 (2875.5 cm^−1^) and -COO- (1186.0 cm^−1^) peaks, indicating potential hydrogen bonding. The unchanged kaolin interlayer -OH peak at 3619.9 cm^−1^ suggested that only surface-active groups were involved. The XRD patterns (Figure 2B) showed that kaolin’s characteristic peaks were present in all the DTG-K samples, which became more pronounced as the kaolin content increased. Additionally, the broad peak at 20.9° shifted to 21.3°, likely due to the kaolin disrupting the tertiary structure of the gel and forming hydrogen bonds with -NH2 groups [21].

### 2.2. Physical Property Characterization Results

As shown in Figure 2C, the water absorption capacity of the DTG-K powders was evaluated. Compared to the pure DTG system (water absorption: 234.8%), the DTG-K series exhibited a positive correlation between water absorption and kaolin content, with DTG-K15 demonstrating the highest absorption rate of 240.7%. This difference was statistically significant compared to DTG (n = 3, *p* < 0.05). Figure 2D displays the swelling ratio curves of the DTG-K powders. Throughout the swelling process, the maximum swelling ratio was attained by all materials within 1 h and exhibited a positive correlation with kaolin content, with DTG-K15 reaching the highest value of 6%. This was followed by a gradual decline in the swelling ratios in all the materials as the immersion time increased. Quantitative pore size analysis was performed using Nano Measurer (v1.2) software on the SEM images shown in Figure 2E (n > 100). The results (Figure S4, Supplementary Materials) demonstrated a progressive decrease in the average pore diameter from 2.02 ± 0.97 μm (DTG) to 1.34 ± 0.38 μm (DTG-K15) with increasing kaolin content, accompanied by improved pore uniformity.

As shown in Figure 2F, DTG-K15 exhibited the highest initial mass loss of 6.03% during the early stage (1–4 days), which was 1.23-fold higher than that of DTG (5.44%). In contrast, during the mid-to-late stages (7–21 days), DTG-K10 and DTG-K15 demonstrated enhanced stability, with DTG-K15 showing a lower cumulative mass loss (19.02%) compared to DTG (30.24%) on day 21. As shown in Figure 2G, the adhesion strength of DTG-K on the pigskin was tested using the overlap–shear method. The pigskin was fixed to a polyimide (PI) sheet; DTG-K-derived gel was applied to two degreased pigskin samples, followed by multiple adhesion tests. The adhesion strength of DTG-K showed significant composition dependence (Figure 2H). Compared to the pure DTG system (maximum adhesion strength of 20.2 kPa), kaolin doping significantly enhanced interfacial adhesion, with DTG-K15 achieving 58.3 kPa, an 189% increase over DTG. The enhancement showed a nonlinear growth trend with increasing kaolin content: DTG-K1, DTG-K2, DTG-K5, and DTG-K10 showed increases of 33.3%, 34.3%, 51.1%, and 119%, respectively. Figure 2I showed the adhesion strength of DTG-K15 in the cyclic adhesion tests: after 12 tensile-adhesion cycles, the strength of DTG-K15 increased from 12.7 kPa to 58.3 kPa; the other DTG-K gels also exhibited similar trends.

### 2.3. Blood Compatibility and In Vitro Coagulation Performance

**Figure 3:**
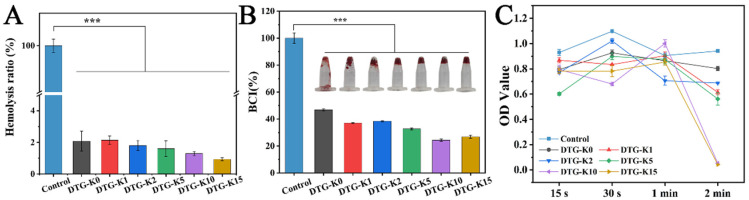
(**A**) Hemolysis ratio of DTG-K materials; (**B**) blood-coagulation indices (BCIs) and blood-coagulation images of DTG-K materials; and (**C**) OD value of the blood-clot supernatant (n = 3, *** *p* ≤ 0.001).

Figure 3A shows the hemolysis test results for DTG-K and the control. In contrast to the Triton X-100 positive control (100% hemolysis), all the DTG-K groups exhibited hemolysis rates below the safety threshold of 5% for blood materials [22].

The blood-clotting indices (BCIs) of the DTG-K materials (Figure 3B) showed significant differences compared to the control group (*** *p* ≤ 0.001, n = 3). The BCIs of the DTG-K materials ranged from 47% (DTG-K0) to 24% (DTG-K10).

**Figure 4:**
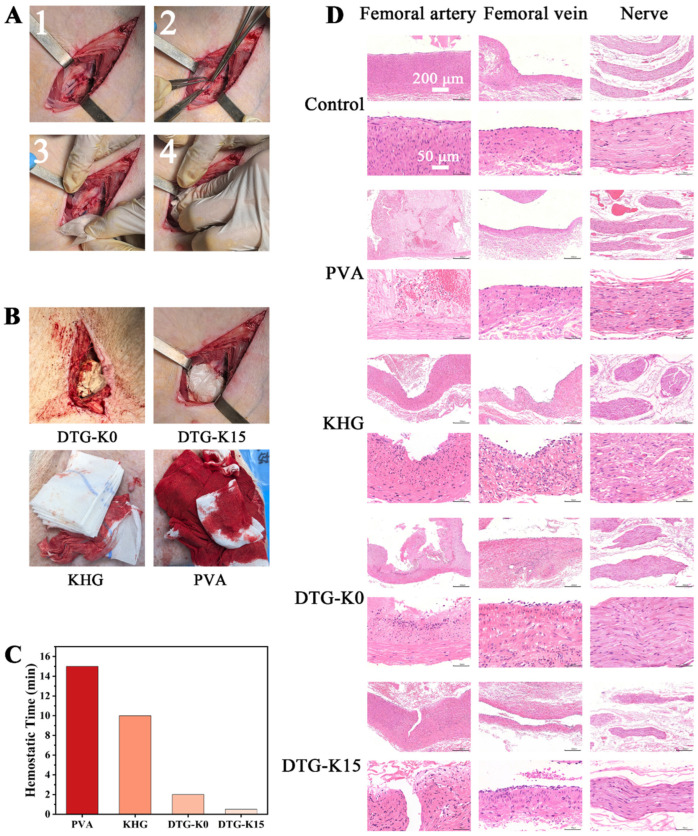
(**A**) Experimental procedure for hemostasis experiment in Bama swine femoral artery hemorrhage model; (**B**) hemostatic effects of materials; (**C**) hemostatic time of materials in the Bama swine femoral artery hemorrhage model; and (**D**) H and E staining photomicrographs of postoperative tissues.

As shown in Figure 3C, the optical density values (OD values) at 540 nm for the supernatant of each material group were measured. Lower OD values indicate less unclotted blood and a better clotting ability [23]. Within the first minute, the blood clots formed by all the materials were easily dispersed by PBS, with no significant coagulation observed for any group. At 2 min, a “threshold effect” similar to the BCI results was observed, with DTG-K10 and DTG-K15 showing significantly lower OD values than the other groups, indicating rapid clotting within 2 min for whole blood. The images in Figure 4B show the results of the 15 s blood-agglutination experiment. The control group failed to form a clot within 15 s, while the blood in the DTG and DTG-K groups had already clotted.

### 2.4. In Vivo Hemostatic Performance of DTG-K in Bama Pig Femoral Artery Models

Before the femoral artery hemostasis experiment, we recognized that the high-pressure and fast blood flow might flush the powdered DTG-K material, making it hard to retain the material near the arterial wound, causing hemostasis failure. Therefore, this study used a medical-grade polyvinyl alcohol (PVA) material, which was molded into a dissolvable bag, to encapsulate the DTG-K materials. This bag dissolves upon contact with water, releasing the internal DTG-K powder into the wound site. Based on the results of pilot experiments, we chose to cut the bag in a cross shape before the start of the experiment to directly expose the internal powder to damaged blood vessels, tissues, and blood. This minimized the impact of the PVA bags on hemostasis, adhesion sealing, and subsequent implantation. An additional control group with equal-weight fragments of PVA dissolvable bag material was also included. Different compression times (10 s, 20 s, 30 s, 40 s, 50 s, 1 min, 2 min, 3 min, and 4 min) were systematically implemented to evaluate the hemostatic efficiency of the materials.

Figure 4A shows a schematic of the hemostasis experiment in the Bama pig femoral artery hemorrhage model. Commercial kaolin hemostatic gauze (KHG) was used as a control group; it exhibited poor initial hemostatic performance, requiring three pieces of KHG and about 10 min to achieve hemostasis, with a blood loss of approximately 20.9 g. The PVA dissolvable bag group also required two additional bags and took about 15 min to stop bleeding while losing about 153.2 g of blood (Figure 4C). Furthermore, after removing the KHG and PVA hemostatic material, it was found that the wound was still bleeding, indicating that PVA cannot stop femoral artery hemorrhage (Figure 4B). Compared to the control groups, the DTG-K materials consistently achieved hemostasis within 2 min, demonstrating significantly superior hemostatic efficiency (Video S1, Supplementary Materials). Notably, DTG-K15 exhibited the shortest hemostasis time of 30 s. After hemostasis, no obvious adhesion between the hemostatic material bag and surrounding tissues was observed; no excess blood or clots were present (Figure 4B). Postoperatively, when the excess hemostatic material was removed, the residual gel-like DTG-K was directly in contact with the arterial wound. This residual DTG-K continued to adhere and seal the vascular rupture, effectively preventing rebleeding. The pulsation of the artery could still be palpated, indicating that the material did not interfere with blood flow and was effective in inducing hemostasis (Video S2, Supplementary Materials).

### 2.5. Postoperative Tissue-Staining Observation

The results of the histological analysis revealed distinct tissue responses across groups (Figure 4D). Arterial sections in KHG and DTG-K0 groups exhibited vascular wall degeneration/necrosis with inflammatory infiltration and thrombosis, whereas the blank controls showed intact morphology. Venous specimens displayed vascular wall degeneration in KHG/DTG-K0 groups but endothelial hyperplasia in DTG-K15. Neural tissues demonstrated axon–myelin separation (KHG group) and nerve fiber congestion (PVA bag group), while other material-treated groups retained normal neural architecture [24,25].

**Figure 5:**
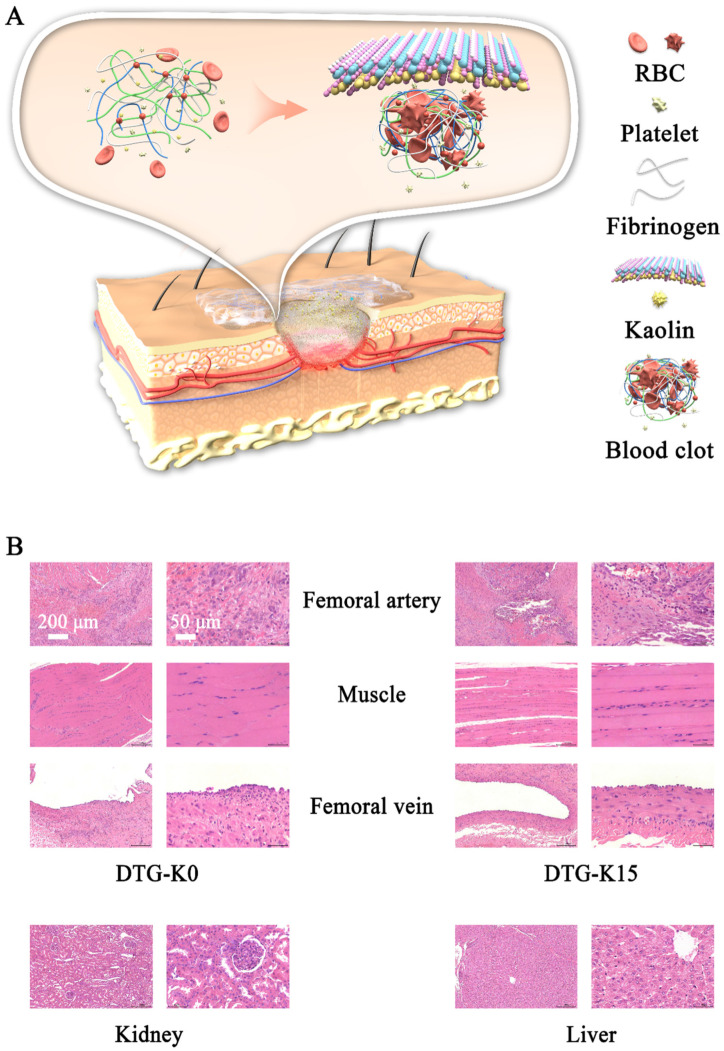
(**A**) Schematic of hemostatic mechanism of DTG-K; and (**B**) H and E staining images of the wound treated with DTG-K0 and DTG-K15 on day 7.

During the embedding period, the experimental pig did not show signs of depression, reduced food intake, or death. Due to femoral artery modeling on both sides, its movements were somewhat restricted; however, it could still stand and walk normally. After seven days, the wounds on both sides returned to normal (Figure S5, Supplementary Materials). Upon reopening the wounds, apart from small amounts of undegraded DTG-K gel; no oozing, hematoma, or blood clots were observed. H and E staining images of the arterial tissue treated with DTG-K0 and DTG-K15 (Figure 5B) showed similar histological changes, including arterial wall degeneration/necrosis, inflammatory cell infiltration, thrombus organization, and perivascular fibrous tissue proliferation, with no significant differences between groups. In the vein tissue, the DTG-K0 group showed endothelial cell hyperplasia and inflammatory cells. Both the DTG-K0 and DTG-K15 groups showed no significant muscle tissue damage. Additionally, the kidney and liver tissues exhibited a clear structural organization without notable pathological changes. Overall, except for slight damage in the vein in the DTG-K0 group, no significant damage was observed in the veins and muscles; DTG-K0 and DTG-K15 did not cause significant pathological changes in the kidneys and livers of the Bama pig.

## 3. Discussion

The results of FTIR and ^1^H NMR confirmed that DP, a catechol-terminated alternating copolymer, was formed through the Michael addition reaction between C=C bonds from PEGDA and the amino groups from DA. Then, DP’s catechol groups formed a multi-hydrogen-bonded crosslinked network with the gel’s amino/carboxyl groups via TA, created a biomimetic adhesive (DTG, derived from the initials of DP, TA, and gel). Kaolin, a natural clay mineral, has a multi-layered silicate structure with abundant hydroxyl groups and metal ions on its surface, which can enhance the cohesion of DTG through hydrogen bonding or metal chelation. XRD and FTIR results (Figure 2A,B) showed that when DTG-K (derived from DTG and the initial of Kao) was rehydrated into a gel, kaolin embedded into DTG’s network formed hydrogen bonds with functional groups such as -NH2, thus acting as a mechanical anchor in the network rather than just as a physical filler.

As a hemostatic material, strong absorption capability facilitates blood concentration and thrombus formation. To evaluate the material’s capacity to rapidly uptake liquid components within a short time frame (0–10 min), the water absorption capacity of the DTG-K powders was tested; Figure 2C shows the results. The DTG-K series exhibited a positive correlation between the water absorption and kaolin content. During water infiltration, the outer layer of the gelatin particles exhibits a higher water absorption rate, while their hierarchical three-dimensional structure unfolds slowly in aqueous environments, thereby hindering further internal moisture penetration. This phenomenon was also observed in the DTG particles. In the DTG systems, the surface gelatin preferentially formed a hydrated barrier upon gelation, necessitating slow external water permeation (permeation rate < 0.14 mL/min) to complete internal gelation. The incorporation of kaolin created water transport channels, overcoming the kinetic limitations imposed by the gelatin barrier. Consequently, the permeation rate of DTG-K15 increased to 0.18 mL/min.

Excessive swelling typically compromises material cohesion and weakens interfacial adhesion. However, the kaolin integration exhibited dual effects. On the one hand, its intrinsic hydrophilicity enhanced the water absorption and retention capacities. On the other hand, the hydrogen-bonding interactions between kaolin and DTG acted as internal anchoring points, effectively suppressing the over-swelling of the DTG network. This antagonistic mechanism resulted in controlled micro-swelling behavior in the DTG-K materials, which not only maintained cohesion through anti-swelling properties but also preserves strong tissue adhesion in wet environments, making them suitable for high-pressure hemorrhage management. The SEM images (Figure 2E) of DTG-K show more detailed structures. The interconnected porous architecture allows for the DTG-K to absorb water rapidly. As the kaolin proportion increases, the material’s pore networks become smaller and more uniform. These phenomena occurred because the kaolin formed anchoring nodes with the DTG network and prevented the DTG polymer network from expanding excessively when it came into contact with water.

The in vitro degradability of DTG-K was also tested. The early (1–4 days) degradation behavior shown in Figure 2F is mainly attributed to the preferential detachment of loosely bound surface kaolin particles. However, in the late stage of degradation (7–21 days), DTG-K15 showing a lower cumulative mass loss than DTG. As mechanically anchored points within the DTG network, kaolin effectively dissipated mechanical stress and slowed the disintegration of the DTG network caused by the hydrolytic cleavage of DP ester bonds. This finding is consistent with the FTIR and XRD results. The kaolin shed in the early stages can rapidly interact with coagulation factors in blood to trigger the coagulation cascade. This action, combined with the sealing function of the biomimetic adhesive to block wounds, works synergistically to achieve hemostasis and promote faster blood-clotting. Later, the polymer network maintains high structural integrity in a body-fluid environment and the kaolin layers embedded in the DTG network can dissipate mechanical stress through dynamic hydrogen bonding.

**Figure 6:**
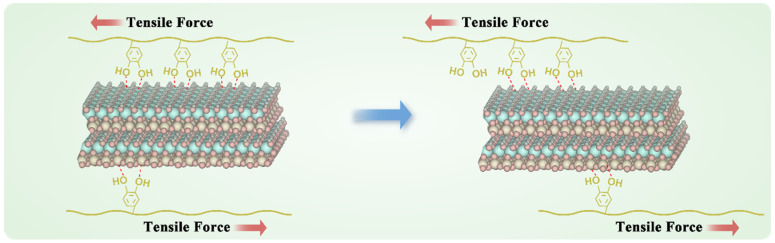
Schematic illustrating the inhibitory effect of kaolin on DTG polymer chain slippage.

The enhancement of adhesion strength shows a nonlinear growth trend with increasing kaolin content (Figure 2G). This supralinear enhancement arose from the synergistic interaction between the kaolin and DTG. The kaolin layers not only acted as rigid fillers to enhance mechanical strength but also formed a 3D hydrogen-bonding network with DTG’s -NH_2_, -COOH, and catechol groups, inhibiting polymer chain slippage (Figure 6). This slippage suppression was more pronounced in the cyclic adhesion test; the adhesion strength of DTG-K increased with the number of tensile adhesion cycles. Initially, kaolin is randomly distributed in the DTG gel, forming primary anchoring points. The high water content (>230%) gives DTG-K viscoelastic properties that lean towards fluidity. As water evaporates during cycling and particles are directionally rearranged by mechanical stress, kaolin becomes enriched at the interface, increasing the density of mechanical anchors per unit volume, resulting in a more ordered polymer–kaolin layered sliding effect and a denser adhesion interface. This “stress-driven” mechanical strengthening effect allows for the materials to realign internal kaolin particles under repetitive mechanical stress (e.g., pulsatile bleeding, vibrations during transport, or the mechanical stretching of wounds), gradually achieving maximum adhesion strength and impact resistance, ensuring stable adhesion and sealing of the wounds during medical evacuation.

Blood compatibility is one of the essential properties of hemostatic materials. The hemolysis test results exhibited good blood compatibility of DTG-K (Figure 3A). Although no statistically significant differences were observed among the DTG-K groups, a trend of a decreasing hemolysis rate with increasing kaolin content was noted, with the DTG-K15 group exhibiting a hemolysis rate of only 0.93%.

The BCI results showed the coagulation capability of the DTG-K materials (Figure 3B). When the kaolin content was low (1–2%), the enhancement in the coagulation ability was limited. However, when the kaolin doping ratio reached a threshold of 5–15%, the improvement in coagulation performance was significantly different from DTG, with the differences in BCI becoming more pronounced as the kaolin content increased. The kaolin in DTG-K not only interlocks with the DTG network through hydrogen bonding to enhance the material’s cohesion, but can also activate coagulation factor XII, thereby accelerating the clotting process. The 15 s blood-coagulation overturning test images in Figure 3B confirmed these results. The control group failed to form a clot within 15 s, while the DTG-K groups, due to their viscosity and ability to absorb plasma and form a gel, maintained the clot’s structural integrity. To further analyze the coagulation process of the DTG-K materials, the coagulation performance was evaluated at four time points: 15 s, 30 s, 1 min, and 2 min (Figure 3C). The “threshold effect”, similar to the BCI results, was observed at 2 min, with DTG-K10 and DTG-K15 showing significantly lower OD values than the other groups, indicating rapid clotting within 2 min for whole blood.

The blood-coagulation process mediated by the DTG-K materials can be divided into two phases. Initially, during the concentration and aggregation phase, kaolin accelerates the absorption of liquid blood components by DTG-K, concentrating red blood cells and promoting their aggregation on the material’s surface. Simultaneously, DTG-K undergoes gelation to form a viscous layer, establishing primary adhesion with blood cells. Subsequently, during the synergistic coagulation phase, kaolin activates factor XII, driving the conversion of fibrinogen to fibrin. This fibrin forms a matrix that envelops the aggregated blood cells on the material’s surface and interpenetrates the DTG-K crosslinked network, resulting in coordinated hemostasis.

In order to evaluate the in vivo hemostatic ability of the DTG-K material, we performed hemostasis experiments on Bama pig femoral artery models (Figure 4A). As shown by the hemostatic performance of the materials (Figure 4B,C), PVA itself lacks the ability to seal arterial wounds and achieve hemostasis. Commercial kaolin hemostatic gauze (KHG), despite containing kaolin and having potential procoagulant effects and a tightly woven structure that can impede red blood cell flow, still allowed blood leakage from gaps between the gauze and wound under high femoral artery flow rates; that is, the KHG showed no sealing function. In contrast, the exceptional hemostatic performance of DTG-K15 is likely attributable to its unique physical and chemical properties, which facilitate rapid wound adhesion and sealing, thereby reducing blood loss. First, the DTG-K materials enclosed in a PVA dissolvable bag had shaping advantages. As shown in Figure 5A, the powdered form of DTG-K imparted moldability, allowing it to conform to the shape of the wound even when packaged in a PVA bag during the wound-packing and sealing process. Upon absorbing water and forming a gel, it can firmly and tightly adhere to the surface of the wound tissue, effectively sealing the damaged blood vessels and the wound. Moreover, the kaolin contained in the DTG-K activated the coagulation mechanism, enabling the DTG-K network to interact synergistically with fibrinogen, red blood cells (RBCs), and platelets to form a blood clot. This adhesion–coagulation synergy facilitated rapid hemostasis.

To evaluate the acute effects of the materials on damaged arteries and the surrounding tissues, samples were collected from the arteries, adjacent veins, and nerves of the control and one of the DTG-K material group pigs (Figure 4D). The arterial lesions in KHG/DTG-K0 groups were likely due to surgical trauma and prolonged hemostatic compression. The absence of neural abnormalities in the material-treated groups (except PVA control) implied that the DTG-K biomaterials exhibit favorable biocompatibility without inducing acute neurotoxicity [24,25]. The H and E staining results of tissue sections after 7 days of embedding are shown in Figure 5B; except for minor vein damage on the DTG side, neither significant vein and muscle damage nor significant pathological changes in the kidney and liver of Bama pigs were observed. During the embedding period, the experimental pig showed no abnormal physiological phenomena except for a slight limitation of movement. These results demonstrated that the small amount of DTG-K0 or DTG-K15 material residues at the wound sites would not cause serious effects, showed the favorable biocompatibility profiles of DTG-K.

## 4. Materials and Methods

### 4.1. Materials

Poly (ethylene glycol) diacrylate (PEGDA, Mn = 600), dopamine hydrochloride (DA), dimethyl sulfoxide (DMSO), triethylamine (TEA), tannic acid (TA), kaolin (Kao), and methyl tertiary butyl ether (MTBE) were purchased from Aladdin Industrial Corporation (Shanghai, China). The gelatin (gel, Mn = 50 000) was obtained from Sigma-Aldrich (Shanghai, China).

### 4.2. Preparation of Biomimetic Adhesive/Kaolin Material Powders

Based on references [16,17,26,27,28], polymers with adhesive groups (DP, derived from the initials of DA and PEGDA) were synthesized via the Michael addition reaction: dopamine hydrochloride (DA; 11.4 g, 60 mmol) and polyethylene glycol diacrylate (PEGDA; 36 g, 60 mmol) were dissolved in 90 mL of dimethyl sulfoxide (DMSO) and deoxygenated under nitrogen protection. The pH was adjusted to 7.9–8.1 with triethylamine (TEA), and the mixture was stirred at 80 °C for 2 h. Excess DA was then added to continue the reaction for another 1 h to cap the vinyl groups. The reaction mixture was washed five times with methyl tert-butyl ether (MTBE) and vacuum-dried to obtain a pale yellow, transparent liquid DP. All reactions were protected from light, and the product was stored at 4 °C in the dark.

The gelatin (gel, 20 g) was dissolved in 200 mL of deionized water at 45 °C to prepare a 10% (*w*/*v*) solution. Tannic acid (TA, 40 g) was dissolved in 200 mL of deionized water to prepare a 20% (*w*/*v*) solution. The DP, TA, and gel were mixed in a volume ratio of 3:2:8 under rapid stirring to form a biomimetic adhesive gel (DTG, derived from the initials of DP, TA, and gel). The mixture was washed with deionized water to collect the adhesive. The DTG gel was frozen at −20 °C, lyophilized, and ground into a powder. The DTG powder was mixed with kaolin (Kao) at mass ratios of 0%, 1%, 2%, 5%, 10%, and 15% to obtain biomimetic adhesive/kaolin composites (DTG-K, derived from DTG and the initial of Kao). All materials were stored in a sealed, moisture-proof environment.

### 4.3. Chemical Characterization

^1^H NMR spectra of PEGDA, DA, and DP were obtained using a nuclear magnetic resonance (NMR) spectrometer (AV II-400 MHz, Bruker, Billerica, MA, USA) with deuterated dimethyl sulfoxide (DMSO-d6) as the solvent. Fourier-transform infrared (FTIR) spectra were recorded for PEGDA, DA, DP, gel, TA, Kao, DTG, and DTG-K using an FTIR spectrometer (Nicolet 6700, Thermo Fisher Scientific, Waltham, MA, USA) in the wavenumber range of 4000 to 500 cm^−1^. To highlight the interactions between the biomimetic adhesive and kaolin in DTG-K, the materials were dissolved in deionized water, stirred to restore a gel-like state, lyophilized again, and ground into a powder. The X-ray diffraction (XRD, D/Max-250, Rigaku Corporation, Tokyo, Japan) patterns of the kaolin and lyophilized DTG-K powders were obtained in a scanning 2θ range of 5° to 60°.

### 4.4. Fluid Absorption Ratio

The mass of an empty centrifuge tube was first measured and designated as G_0_. After the addition of approximately 1 g of a DTG-K powder, the total mass was then recorded as G. Subsequently, 10 mL of deionized water was added, and the mixture was oscillated at 37 °C for 10 min. The unabsorbed liquid was removed, after which, the total mass was measured and recorded as G_t_. Triplicate experiments were conducted. The water absorption rate was calculated using the following formula: water absorption (%) = (G_t_ − G_0_)/(G − G_0_) × 100%. The permeation efficiency during the water absorption process was calculated as the water absorption rate divided by the absorption time.

### 4.5. Swelling Ratio

The swelling ratio test was performed according to methods used in the literature [29]. The initial mass of the material was weighed and designated as W_0_, after which it was immersed in PBS (pH 7.4). The system was oscillated at 37 °C; the surface liquid was periodically removed at specified intervals (1–24 h) to measure the swollen mass (W_t_). The swelling ratio was calculated using the following formula: swelling ratio (%) = (W_t_ − W_0_)/W_0_ × 100%.

### 4.6. Structural Characterization of DTG-K-Derived Gel

After the DTG and DTG-K materials were fully hydrated, crosslinked, and lyophilized, their microstructures were observed using a scanning electron microscope (SEM; JSM-5600LV, JEOL, Tokyo, Japan). Quantitative pore size analysis of the SEM images (n > 100) was performed using Nano Measurer (v1.2) software.

### 4.7. In Vitro Degradability

Following the method used in reference [30], the DTG and DTG-K samples (M_0_) were immersed in phosphate-buffered saline (PBS) at a 1:30 (*w*/*v*) ratio. The mixture was oscillated at 37 °C and 80 rpm, with the PBS replaced every 3 days. Samples were removed at predetermined time points (1–21 days), vacuum-dried at 60 °C to a constant weight (M_t_), and the mass loss was calculated using the following formula: mass loss (%) = [(M_0_ − M_t_)/M_0_] × 100%.

### 4.8. Adhesion

Overlap–shear tests were performed as described in reference [18]. Fresh pigskin was defatted by washing it three times with 70% isopropanol, alternating with anhydrous ethanol, and then hydrated with saline and covered with gauze. The pigskin was cut into 20 mm × 30 mm pieces; the subcutaneous side was fixed to a 20 mm wide piece of polyimide (PI) film using commercial cyanoacrylate adhesive, followed by curing for 5 min. The DTG-K powder was mixed with water at a 1:2 (*w*/*v*) ratio to form a glue, which was applied to the pigskin surface (20 mm × 30 mm bonding area) and cured for 5 min. Another sample was overlapped and pressed uniformly for 5 min. Using a universal testing machine (AGS-X, Shimadzu, Kyoto, Japan), the samples were pulled apart at 100 mm/min until complete separation occurred. The maximum load was recorded and used to calculate the adhesion strength (maximum load/bonding area). Multiple tests were performed on the same sample after re-adhesion.

### 4.9. Hemocompatibility Assay

The hemolysis ratio was measured according to that used in references [30,31]. Detoxified samples (10 mg) were incubated in 2 mL of PBS at 37 °C for 24 h, centrifuged, and the supernatant was collected. New Zealand rabbit whole blood (3.2% sodium citrate) was centrifuged at 1200 rpm for 10 min to separate the blood cells, washed thrice with PBS, and diluted to form a 5% suspension. A 500 μL volume of the sample supernatant was mixed with an equal volume of the blood cell suspension and incubated at 37 °C for 1 h. After centrifugation at 1200 rpm for 5 min, the absorbance of the supernatant at 540 nm was measured using a microplate reader (n = 3). Triton X-100 (0.1%) and PBS were used as the positive and blank controls, respectively. The hemolysis ratio was calculated using the following formula: hemolysis ratio (%) = [(A_Sample_ − A_PBS_)/(A_Triton_ − A_PBS_)] × 100%.

### 4.10. In Vitro Blood-Clotting

As per references [32,33], detoxified samples (10 mg) were mixed with 20 μL of CaCl_2_ (0.2 M) and 200 μL of anticoagulated blood, vortexed, and incubated at 37 °C for 3 min. After adding 10 mL of PBS and oscillating at 80 rpm for 10 min, 100 μL of the supernatant was taken to measure its absorbance at 540 nm using a microplate reader (Multiskan FC). Groups without any materials were used as the controls, with three parallels per group. The blood-clotting index was calculated as follows: BCI (%) = (A_i_/A_0_) × 100%, where A_i_ and A_0_ are the absorbances of the material and control groups. Similarly, samples (10 mg) were mixed with 200 μL of an anticoagulated blood–CaCl_2_ mixture (10:1, *v*/*v*), incubated at 37 °C for 15 s, 30 s, 1 min, and 2 min, then suspended in 10 mL of PBS. A 100 μL volume of the supernatant was taken to measure its absorbance at 540 nm (n = 3). Additionally, 200 μL of the anticoagulated blood–CaCl_2_ mixture (10:1, *v*/*v*) was added to a centrifuge tube containing 50 mg of the material; the clotting effect was recorded by inverting and photographing after 15 s. A centrifuge tube without any material was set as the control group.

### 4.11. Femoral Artery Hemostasis

The animal study protocol was reviewed and approved by the Ethics Committee of Sichuan Lilaisinuo Biological Technology Co., Ltd. (Chengdu, China, ethical approval number: LLSN-2024329). As per reference [34], three healthy Bama pigs (15–20 kg, female) were randomly assigned to two test material groups (two pigs each) and one control material group (one pig). A bleeding model was established in the bilateral femoral arteries, with hemostasis performed using the test materials (DTG-K0 and DTG-K15) and control materials (kaolin hemostatic gauze and PVA dissolvable bags). After 7 days of acclimatization (16–26 °C, 30–70% humidity, and 12-h light cycle), pigs with no physiological abnormalities were selected for the experiment. Preoperatively, the pigs were fasted for 12 h and received intramuscular injections of buprenorphine (0.025 mg/kg) for analgesia and glycopyrrolate (0.01 mg/kg) to suppress vagal nerve reactions. Anesthesia was induced with 5% isoflurane, followed by endotracheal intubation and mechanical ventilation (maintaining the end-tidal CO_2_ at 40 ± 2 mmHg). The pigs were placed in a supine position; the inguinal region was shaved and disinfected. A 10 cm incision was made to expose 5 cm of the femoral artery, which was clamped proximally and distally with hemostatic forceps. A 4 mm longitudinal incision was made in the artery; any residual blood was cleared and blood flow was restored. Immediately, 3 g of the test/control material in polyvinyl alcohol (PVA) dissolvable bags were applied to the wound; medical gauze was used to absorb any exudate. Pressure was applied to the wound; hemostasis time, blood loss, and imaging data were recorded simultaneously. Hemostasis was considered effective if no blood leakage, material bag detachment, or rebleeding was observed within 1 min after pressure release. The total hemostasis time was defined as the duration from initial compression to pressure release.

### 4.12. Histological Analysis

The control and one of the test material group animals were euthanized with an overdose of anesthesia postoperatively. The injured artery, adjacent vein, and nerve tissue were harvested for fixation, embedding, sectioning, and H and E staining. The other material group animal underwent wound debridement and suturing (alternating disinfection with medical ethanol and iodine) and was monitored for one week for food intake, mobility, and wound healing. As a pre-hospital emergency arterial hemostatic material, DTG-K should be completely removed upon hospital admission, followed by replacement with standard clinical hemostatic interventions. To evaluate the long-term effects of minor residual material at the wound site, all materials involved in the hemostatic process were intentionally retained prior to wound closure. On postoperative day 7, tissues from the tested artery, adjacent vein, muscle, liver, and kidneys were collected for fixation, embedding, sectioning, and H and E staining. Tissue inflammation, vascular injury, and organ lesion severity were assessed using H and E staining combined with a four-grade scoring method.

### 4.13. Statistical Analyses

All data are presented as the mean ± standard deviation (M ± SD). Data analysis was performed using one-way analysis of variance (ANOVA). Significant differences were defined as * *p* ≤ 0.05, ** *p* ≤ 0.01, and *** *p* ≤ 0.001, while NS (*p* > 0.05) indicates no significant difference.

## 5. Conclusions

In summary, we used hydrochloric acid dopamine (DA) molecules as the primary source of adhesion, which were copolymerized with polyethylene glycol diacrylate (PEGDA) to form a polymer backbone (DP), which was then combined with tannic acid (TA) and gelatin (gel) through hydrogen bonding to synthesize a biomimetic adhesive (DTG). Subsequently, kaolin was incorporated into the biomimetic adhesive, leading to the development of DTG-K, a material designed for pre-hospital emergency arterial hemostasis. In this system, kaolin and the biomimetic adhesive form multiple synergistic interactions, enhancing adhesion and cohesion to effectively seal arterial wounds and achieve a tight closure for the wound tissue. Additionally, the kaolin activates coagulation factor XII, triggering the coagulation cascade to achieve rapid hemostasis through a dual mechanism of sealing and coagulation. These findings suggest a promising approach to pre-hospital hemostasis; future work should focus on improving its performance and conducting clinical trials.

## Data Availability

The data is unavailable due to privacy.

## Supplementary Materials

The following supporting information can be downloaded at: https://www.mdpi.com/article/10.3390/ijms26104688/s1.
