# Supplementary material for: A Biomimetic Adhesive/Kaolin Material with Strong Adhesion, Sealing, and Active Coagulation Function for Arterial Hemostasis
Source: Int J Mol Sci. 2025 May 14;26(10):4688. doi: 10.3390/ijms26104688 (PMC12111511; doi:10.3390/ijms26104688)
Supplement: Supplementary file 1 [file ijms-26-04688-s001.zip › ijms-3604592-supplementary.pdf]

Article

A Biomimetic Adhesive/Kaolin Material with Strong Adhesion, Sealing, and Active Coagulation Function for Arterial Hemostasis

Wanli Zhang [1,†], Junqin Mao [1,†], Aiping Yang [1], Tao Shen [1], Qiuyu Zeng [1], You Tang [1], Yan Du [2], Guoyu Lv [1],
Heng Zheng [1,*] and Hong Li [1,*]

[1] College of Physics, Sichuan University, Chengdu 610065, China
[2] College of Information Engineering and Automation, Kunming University of Science and Technology, Kunming 650032, China
* Correspondence: zhengheng@scu.edu.cn (H.Z.); lih2013@scu.edu.cn (H.L.)
† These authors contributed equally to this work.

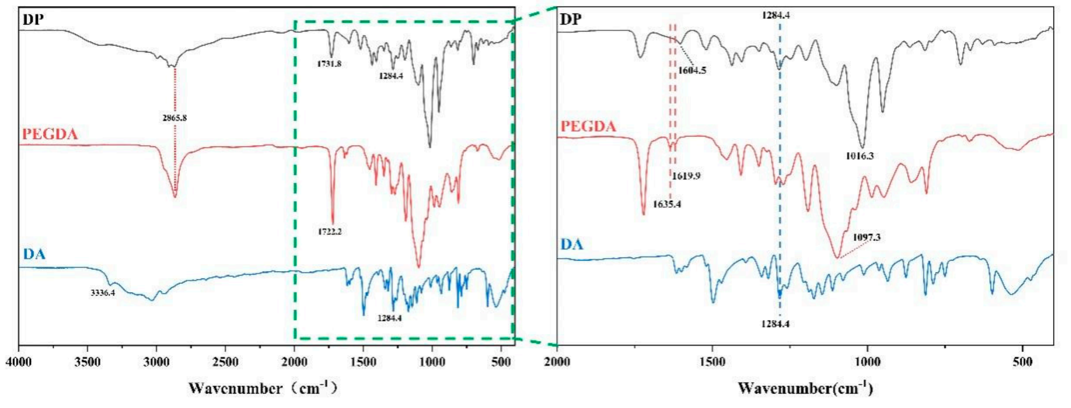

Figure S1 The FT-IR spectra of sample PEGDA、DA and DP.

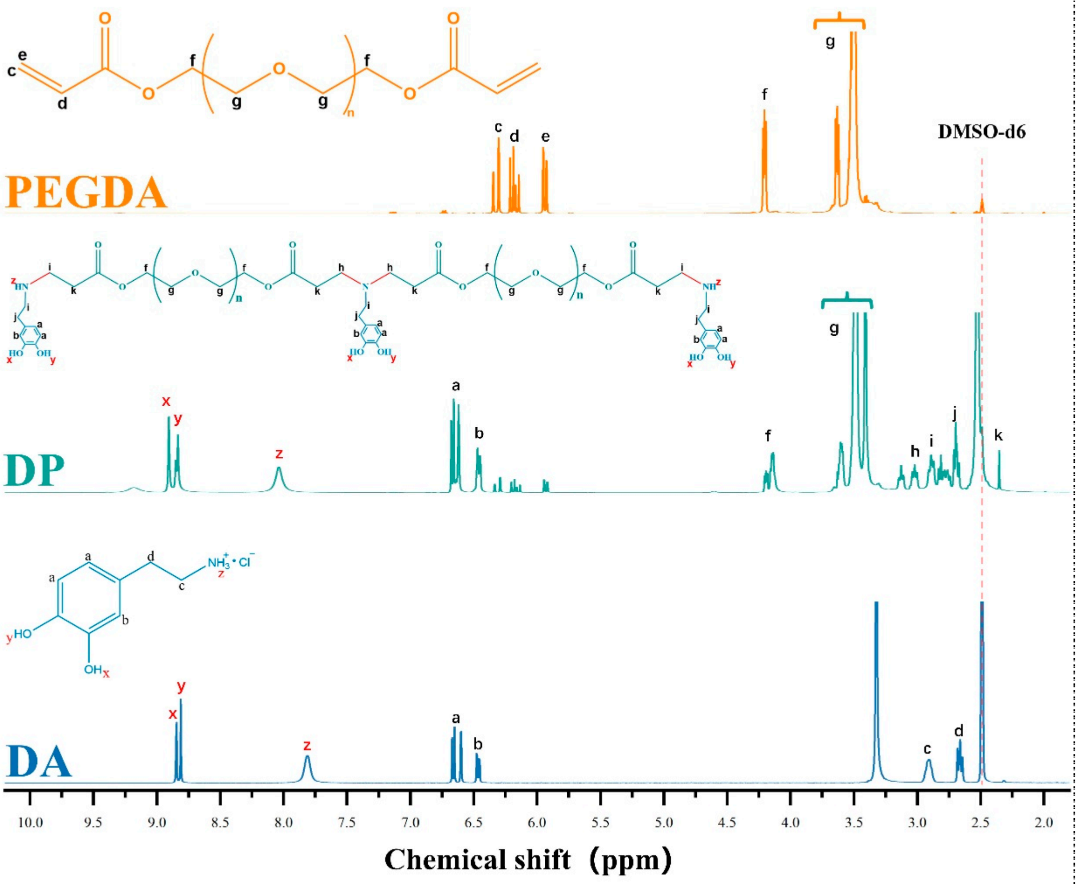

Figure S2 ^{1}H NMR spectra of the sample PEGDA、DA and DP in DMSO-d6.

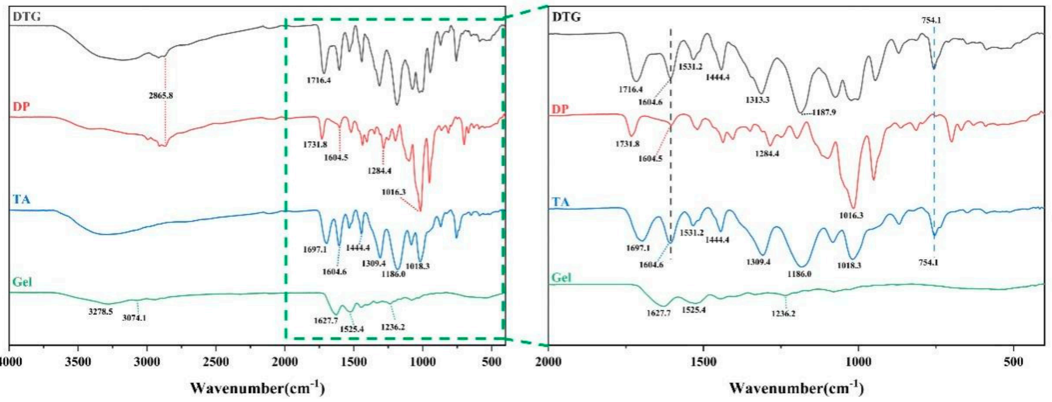

Figure S3 The FT-IR spectra of sample DP、TA、Gel and DTG.

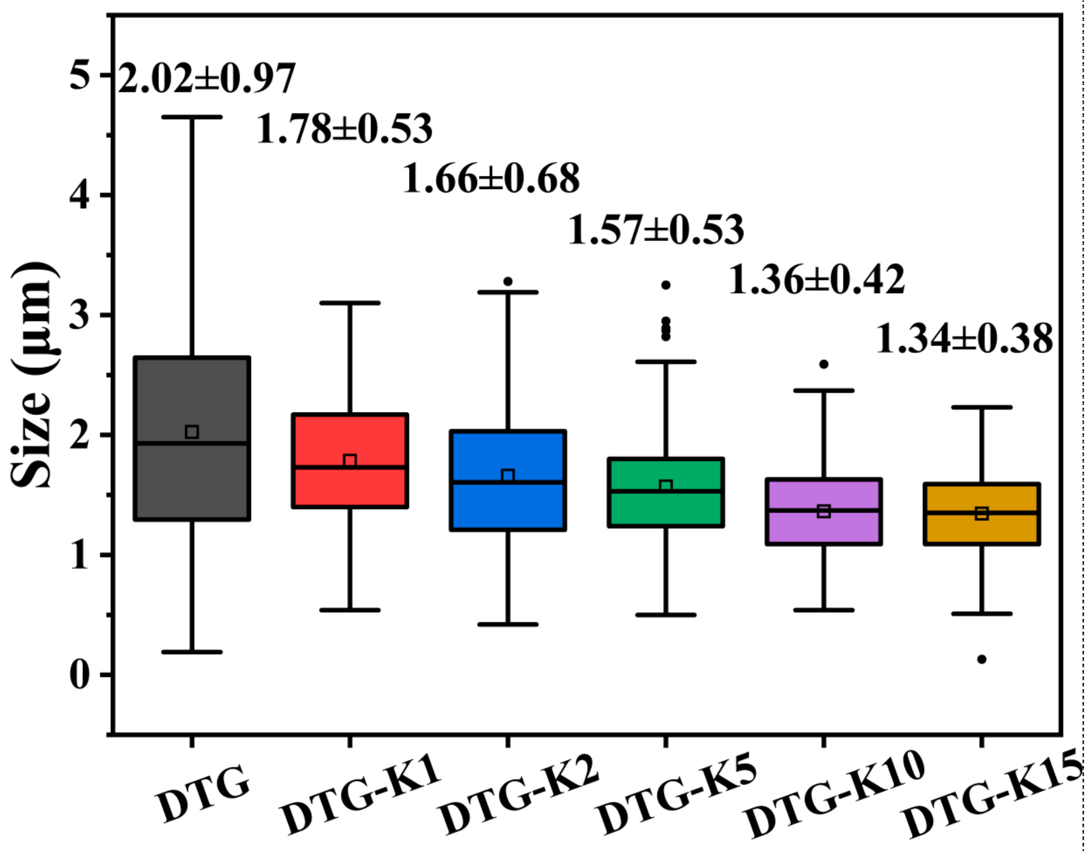

Figure S4 Statistical analysis of pore sizes in SEM images was performed using *Nano Measurer* software.

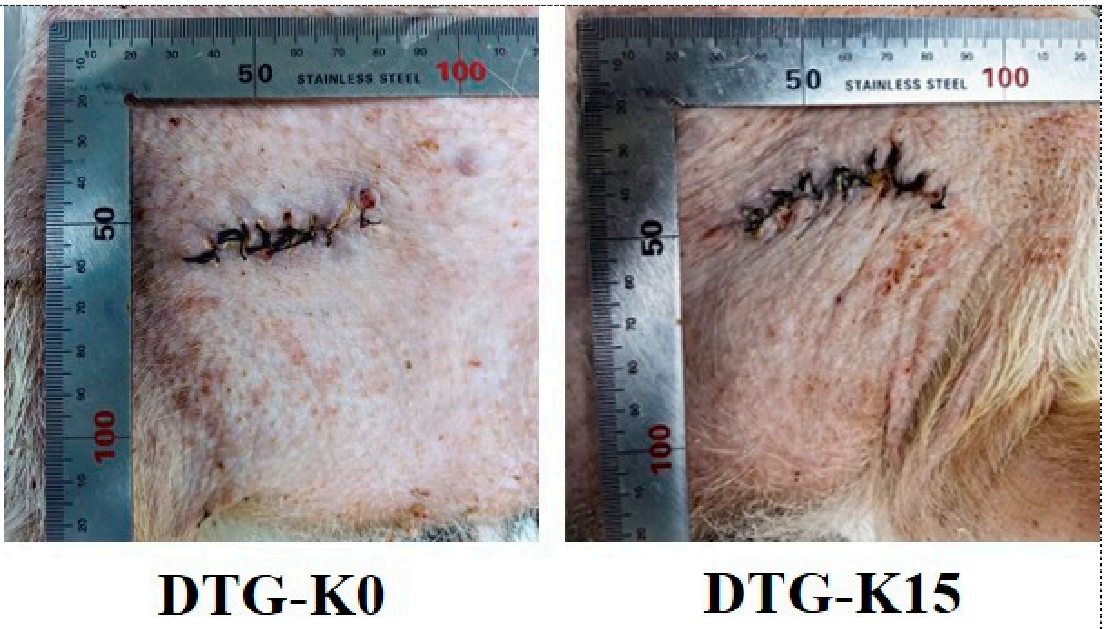

Figure S5 Postoperative wound images on day 7

Videos S1 and S2 can be downloaded and viewed at https://doi.org/10.5281/zenodo.15174273
